# RETRACTED: Shi et al. Effect of Final Rolling Temperature on Microstructures and Mechanical Properties of AZ31 Alloy Sheets Prepared by Equal Channel Angular Rolling and Continuous Bending. *Materials* 2020, *13*, 3346

**DOI:** 10.3390/ma17194921

**Published:** 2024-10-09

**Authors:** Laixin Shi, Lei Liu, Li Hu, Tao Zhou, Mingbo Yang, Yong Lian, Jin Zhang

**Affiliations:** 1 College of Material Science and Engineering, Chongqing University of Technology, Chongqing 400054, China; shilaixin2016@cqut.edu.cn (L.S.); liu_lei0722@163.com (L.L.); yangmingbo@cqut.edu.cn (M.Y.); 2 Institute for Advanced Materials and Technology, University of Science and Technology Beijing, Beijing 100083, China; liany09@126.com (Y.L.); zhangjin@ustb.edu.cn (J.Z.)

The journal retracts the article, “Effect of Final Rolling Temperature on Microstructures and Mechanical Properties of AZ31 Alloy Sheets Prepared by Equal Channel Angular Rolling and Continuous Bending” [1], cited above. 

Following publication, the authors contacted the Editorial Office to raise concerns related to redundancy and the improper reuse of figures from one of their previous articles [2].

Adhering to our standard procedure, the Editorial Office and Editorial Board conducted an investigation that confirmed the following image duplications without the appropriate citation or permission:

Figure 4c in paper [1] is duplicated as Figure 3c in published paper [2].

Figure 7c in paper [1] is duplicated as Figure 6c1 in published paper [2].

Figure 7f in paper [1] is duplicated as Figure 6c2 in published paper [2].

Figure 8c in paper [1] is duplicated as Figure 3f in published paper [2].

The authors previously published Correction [3], which did not include the overlap specified above.

As a result, the Editorial Office, Editorial Board, and authors have decided to retract this article as per MDPI’s retraction policy (https://www.mdpi.com/ethics#_bookmark30, accessed on 8 April 2024).

This retraction was approved by the Editor-in-Chief of the *Materials* journal. 

The authors have agreed to this retraction. 

## References

[1] Shi L.X., Liu L., Hu L., Zhou T., Yang M.B., Lian Y., Zhang J. (2020). RETRACTED: Effect of Final Rolling Temperature on Microstructures and Mechanical Properties of AZ31 Alloy Sheets Prepared by Equal Channel Angular Rolling and Continuous Bending. Materials.

[2] Tu J., Zhou T., Liu L., Shi L., Hu L., Song D., Song B., Yang M., Chen Q., Pan F. (2018). Effect of rolling speeds on texture modification and mechanical properties of the AZ31 sheet by a combination of equal channel angular rolling and continuous bending at high temperature. J. Alloys Compd..

[3] Shi L., Liu L., Hu L., Zhou T., Yang M., Lian Y., Zhang J. (2023). Correction: Shi et al. Effect of Final Rolling Temperature on Microstructures and Mechanical Properties of AZ31 Alloy Sheets Prepared by Equal Channel Angular Rolling and Continuous Bending. *Materials* 2020, *13*, 3346. Materials.

